# Feasibility and acceptability of high-intensity interval training and moderate-intensity continuous training in kidney transplant recipients: the PACE-KD study

**DOI:** 10.1186/s40814-022-01067-3

**Published:** 2022-05-21

**Authors:** Roseanne E. Billany, Alice C. Smith, Ganisha M. Hutchinson, Matthew P. M. Graham-Brown, Daniel G. D. Nixon, Nicolette C. Bishop

**Affiliations:** 1 Department of Cardiovascular Sciences, University of Leicester, Leicester, UK; 2 John Walls Renal Unit, University Hospitals of Leicester NHS Trust, Leicester, UK; 3 Department of Health Sciences, University of Leicester, Leicester, UK; 4 Department of Respiratory Sciences, University of Leicester, Leicester, UK; 5 School of Sport, Exercise and Health Sciences, Loughborough University, Loughborough, LE11 3TU UK

**Keywords:** Kidney transplantation, High-intensity interval training, Cardiovascular disease, Feasibility, Chronic kidney disease

## Abstract

**Background:**

Kidney transplant recipients (KTRs) exhibit unique elevated inflammation, impaired immune function, and increased cardiovascular risk. Although exercise reduces cardiovascular risk, there is limited research on this population, particularly surrounding novel high-intensity interval training (HIIT). The purpose of this pilot study was to determine the feasibility and acceptability of HIIT in KTRs.

**Methods:**

Twenty KTRs (male 14; eGFR 58±19 mL/min/1.73 m^2^; age 49±11 years) were randomised and completed one of three trials: HIIT A (4-, 2-, and 1-min intervals; 80–90% watts at V̇O_2peak_), HIITB (4×4 min intervals; 80–90% V̇O_2peak_) or MICT (~40 min; 50–60% V̇O_2peak_) for 24 supervised sessions on a stationary bike (approx. 3x/week over 8 weeks) and followed up for 3 months. Feasibility was assessed by recruitment, retention, and intervention acceptability and adherence.

**Results:**

Twenty participants completed the intervention, and 8 of whom achieved the required intensity based on power output (HIIT A, 0/6 [0%]; HIITB, 3/8 [38%]; MICT, 5/6 [83%]). Participants completed 92% of the 24 sessions with 105 cancelled and rescheduled sessions and an average of 10 weeks to complete the intervention. Pre-intervention versus post-intervention V̇O_2peak_ (mL/kg^-1^/min^-1^) was 24.28±4.91 versus 27.06±4.82 in HIITA, 24.65±7.67 versus 27.48±8.23 in HIIT B, and 29.33±9.04 versus 33.05±9.90 in MICT. No adverse events were reported.

**Conclusions:**

This is the first study to report the feasibility of HIIT in KTRs. Although participants struggled to achieve the required intensity (power), this study highlights the potential that exercise has to reduce cardiovascular risk in KTRs. HIIT and MICT performed on a cycle, with some modification, could be considered safe and feasible in KTRs. Larger scale trials are required to assess the efficacy of HIIT in KTRs and in particular identify the most appropriate intensities, recovery periods, and session duration. Some flexibility in delivery, such as incorporating home-based sessions, may need to be considered to improve recruitment and retention.

**Trial registration:**

ISRCTN, ISRCTN17122775. Registered on 30 January 2017.

**Supplementary Information:**

The online version contains supplementary material available at 10.1186/s40814-022-01067-3.

## Key messages regarding the feasibility

- What uncertainties existed regarding the feasibility?

There is limited information on adverse events, acceptability, and adherence to high-intensity interval training performed by kidney transplant recipients who may benefit from this type of exercise due to increased cardiovascular disease risk.

- What are the key feasibility findings?

Although participants struggled to achieve the required exercise intensity, this study highlights the potential that exercise has to reduce cardiovascular risk in kidney transplant recipients. Importantly there were no adverse events to any of the exercise protocols and no safety concerns raised.

- What are the implications of the feasibility findings for the design of the main study?

The results of this feasibility study could inform the design, sample size, and appropriate outcome measures to enhance the success of future randomised controlled trials. The results demonstrate a need to examine different high-intensity protocols in order to find the most appropriate and achievable for this population.

## Background

Cardiovascular disease (CVD) is a leading cause of morbidity and mortality after kidney transplantation and a key factor limiting graft survival [1, 2]. Most recent data suggests that for kidney transplant recipients (KTRs), CVD and cerebrovascular disease account for 19.9% of all deaths, making it the second biggest cause of mortality behind malignancy [3]. These mortality rates do not reveal the full impact of the non-fatal cardiovascular events including acute myocardial infarction, heart failure, cardiac arrhythmias, and stroke: [2] all of which limit the survival of the donated kidney and quality of life (QoL) [1].

Traditional CVD predictive modelling underestimates the risk in patients with chronic kidney disease (CKD) suggesting that kidney disease and transplantation introduce unique and exacerbating features not akin to the general population [4]. Annual CVD mortality has been reported as approximately 10 times higher in KTRs aged 25–34 in comparison to the general population of equal age [5]. It is clear that KTRs have an elevated prevalence of traditional [6] and non-traditional [7] CVD risks. Weight gain, obesity, diabetes, hypertension, and metabolic syndrome are prevalent factors in KTRs linked to mortality, graft loss, and cardiac events [6]. Many of these factors are exacerbated by immunosuppressive medications [8, 9]. Unique hemodynamic challenges (anaemia, hypertension, and volume expansion) frequently seen in KTRs can accelerate cardiomyopathy without the concurrent ischaemic heart disease often seen in the general population [2]. These haemodynamic stresses likely contribute to the vascular and systemic inflammation [10] that is paradoxically common in KTRs. Elevations in novel biomarkers of inflammation are thought to not just be indicative but also play a major role in the development of CVD [11, 12]. Dialysis duration, deceased donor transplant, cytomegalovirus infection, and acute rejection episodes have been suggested as CVD risk factors rather than solely threats to graft survival [7].

There is an association between low physical activity (PA) levels and cardiovascular risk [13, 14] and an inverse relationship between PA and all-cause and CVD mortality [15]. Empirical evidence suggests that PA has ‘anti-inflammatory’ effects [11]. Despite this, levels of PA in KTRs remain lower than in the general population [16, 17]; only 27% classify as meeting nationally recommended guidelines [18]. While supervised exercise interventions in KTRs improve cardiorespiratory fitness and a variety of traditional and non-traditional risk factors for CVD, including metabolic profile [19, 20], vascular stiffening [19], weight [21], and inflammation [22], the impact of exercise programmes on hard outcomes in the long term, as well as the best exercise modalities (intensity, duration, and frequency) remain to be adequately addressed [23].

Exercise interventions in KTRs have, to date, focussed on moderate-intensity continuous training (MICT) [24]. Recently, high-intensity interval training (HIIT) programmes have received attention in both the scientific literature and the popular press for providing a unique physiological stimulus that improves traditional and inflammatory markers of cardiovascular risk in both non-clinical and clinical populations to a similar or greater degree than MICT [25], as well as being perceived as more enjoyable [26] and time efficient. Although definitions vary, HIIT is characterised by short bursts of vigorous exercise (≥80% maximal aerobic capacity or 85–95% of peak heart rate (HR_peak_)) interspersed with periods of moderate-intensity exercise or rest. In CKD, HIIT has been associated with improved physical function, inflammation, and QoL in patients receiving peritoneal dialysis [27] and improved cardiorespiratory fitness in patients receiving haemodialysis [28]. It is safe, effective, and well tolerated in patients with CVD and heart transplant recipients [29–31].

Despite the potential benefits of HIIT in KTRs, patients’ views suggest that the strenuous nature may be a barrier to participation [32]. Patient and public involvment by our group has identified that KTRs, particularly those who are active, have expressed interest in HIIT but do not know whether it is safe and do not know how far they can or should “push themselves.” Therefore, the primary aim of this pilot study was to determine the feasibility and acceptability of HIIT in KTRs and the feasibility of progressing to large and complex randomised controlled trials with primary outcomes focussing on reducing cardiovascular risk in this unique population.

## Materials and methods

### Experimental design and participants

A full description of the trial protocol has been published [33]. This report adheres to the CONSORT guidelines for reporting pilot and feasibility trials. The PACE-KD trial was a randomised, three-arm parallel-group study to determine the feasibility and acceptability of three different supervised aerobic exercise programmes. Participants were randomised 1:1:1 to receive 24 sessions of one of two HIIT protocols or MICT across approximately 8 weeks. All groups received usual care alongside the intervention. Inclusion criteria were kidney transplant recipients >18 years old with a stable transplant completed >12 weeks prior to consent. Exclusion criteria were scheduled surgery or procedures involving anaesthesia, pregnancy, significant disease, or disorder which may put the patient at risk while taking part in the study or may influence the results of the study, and the inability to give informed consent or comply with the testing and training protocol. Study assessments were conducted at baseline, mid-training, post-training, and 3 months post-training. Participants were recruited from the University Hospitals of Leicester NHS Trust (UHL; UK) between March 2017 and May 2019, where outcome assessments and exercise training were also conducted.

### Ethical and regulatory details

The University Hospitals of Leicester NHS Trust agreed to act as sponsor for this study on October 31, 2016 (EDGE 88714). East Midlands-Nottingham Research Ethics Committee (REC; ref 16/EM/0482) gave favourable opinion on January 04, 2017. Health Research Authority regulatory approval was given on January 27, 2017, and the study was adopted on the NIHR portfolio on 12/01/2017. The trial was prospectively registered (ISRCTN17122775; January 31, 2017). Local governance approval was granted by UHL Research and Innovation on March 02, 2017. Steps were taken when designing this protocol to minimise the ethical implications and ensure patient welfare. The study complied with the International Conference for Harmonisation of Good Clinical Practice guidelines, the Research Governance Framework for Health and Social Care and was carried out in accordance with the Declaration of Helsinki.

### Randomisation

Following baseline assessments, participants were randomised 1:1:1 to one of the three study groups using computer-generated random numbers (in fixed sized blocks; http://www.randomization.com) stratified by age (≤ 44 or ≥ 45) and estimated glomerular filtration rate (eGFR; ≤ 54 or ≥ 55). The Chief Investigator managed the randomisation and block sizes and allocation sequences were not disclosed.

### Interventions

Participants were invited to attend 24 supervised training sessions over approximately 8 weeks on a stationary cycle ergometer. Each training session was preceded and followed by a 5-min warm-up and 10-min cool-down, respectively. Sessions were supervised by appropriately trained staff, and the heart rate was monitored throughout and guided by the baseline cardiopulmonary exercise results. Details for the intensity and time of each intervention were as follows:

### HIIT A (Fig. 1A)

Each session consisted of a 16-min interval training with intervals of 4-, 2-, and 1-min duration at 80%, progressing to 90%, of watts at oxygen uptake peak (V̇O_2peak_) over the 8 weeks, separated by a 2 min active rest (~60% V̇O_2peak_) giving a total exercise time of 30 min.

**Fig. 1:**
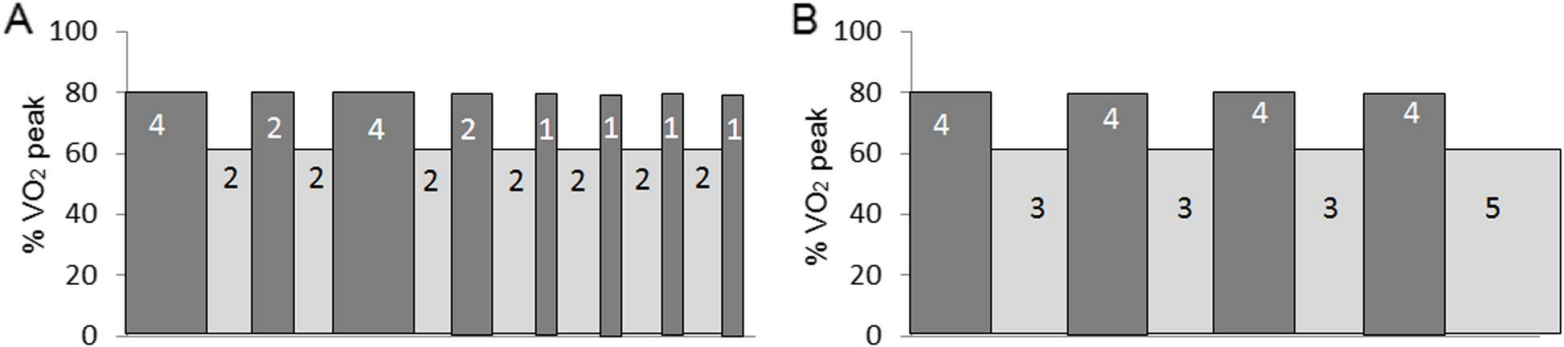
Schematic diagram of HIIT interventions A and B

### HIIT B (Fig. 1B)

Each session consisted of 4×4 min interval training at 80%, progressing to 90%, V̇O_2peak_ over 8 weeks, separated by a 3-min active rest, and final 5-min active stage (both ~60% V̇O_2peak_) to ensure equal overall work done and total session time between both HIIT protocols.

### MICT

Each session consisted of continuous brisk cycling for ~40 min at 50–60% V̇O_2peak_ (rating of perceived exertion (RPE) of 12–14 or ‘somewhat hard’). Time was adjusted in accordance with intensity in order to closely match MICT total external work done with both HIIT protocols.

Secondary to watts achieved, the percentage of HR_peak_ (based on the maximal exercise test) was used to monitor training intensity with the aim of achieving 85–95% of HR_peak_ during the high-intensity bouts and 60% during recovery and for MICT.

### Feasibility and acceptability outcomes

The criterion for success of this feasibility study is based on recruitment, retention, and intervention adherence and acceptability. We revised our previously reported [33] progression criteria in favour of a more pragmatic approach. Specific criteria for progressing to a larger efficacy trial were co-produced between researchers, clinicians, and patients (Table 1) using a condensed version of a previously reported method prior to completion of the study [34]. In short, any value below the specified ‘stop’ criteria and any value above the specified ‘go’ criteria are considered not feasible and feasible to progress to a larger trial, respectively. Any value between ‘stop’ and ‘go’ are considered feasible with modification.

**Table 1:** Progression criteria for PACE-KD

Criteria	Stop	Go	PACE-KD result
The number of patients able to take part (eligibility)	<20%	>50%	60%
The number of patients who are eligible that agree to take part (recruitment)	<20%	>50%	23%^a^
Whether participants achieved^a^ the required exercise intensity during the intervention (intervention acceptability) ^a^at any point during the intervention	<70%	>80%	40%^b^
The number of sessions (out of 24) that participants attended (intervention completion)	<50%	>80%	92%
The numbers of participants who completed all aspects of the trial (trial completion/retention)	<50%	>70%	58%^a^
Whether participants could complete the outcome measures (outcome acceptability; % for completion of one single outcome measure; example V̇O_2peak_)	<60%	>80%	95%

^a^Values between ‘stop’ and ‘go’ can be modified to increase success

^b^75% for secondary HR_peak_ criteria

### Secondary outcomes

The following outcome measures were assessed in all participants at baseline and post-training: cardiorespiratory fitness (via a standard incremental protocol on a stationary cycle ergometer), body composition (via bioelectrical impedance analysis), haemodynamic parameters (via non-invasive cardiac output monitor), physical function (via sit-to-stand, 4-m gait speed, and calf strength), habitual physical activity (via accelerometry), markers of cardiovascular risk, inflammation, and immune function (data to be published elsewhere). Measures were also assessed at mid-training and 3 months post-training with the exception of cardiorespiratory fitness and habitual physical activity. A survey pack containing eight questionnaires was administered at each time point [33].

Participants who completed the intervention were invited to attend a one-to-one semi-structured interview to explore individual perspectives and feelings about the intervention and the trial design (data to be published elsewhere). A participant satisfaction questionnaire (PSQ) was also administered post-training.

A small subset of participants (*n*=15) were invited to receive a multiparametric cardiac MRI (CMR) scan before and after the intervention using previously reported methodology [35–38]. Ten participants consented to take part and seven completed both pre- and post-intervention scans. Data from these participants are combined for the purpose of this report.

### Data analysis

In accordance with guidance on feasibility and pilot studies, no formal hypothesis testing has been undertaken [39]; therefore, quantitative data are summarised using descriptive statistics. Data from paper case report forms were inputted into Microsoft® Excel® then imported into IBM® Statistical Package for Social Sciences 25 (SPSS®) for descriptive analysis and are presented as mean ± SD unless otherwise stated. No formal sample size calculation was performed as the study was pragmatic and not designed to show statistical significance or power. Data collected will allow sample size calculations to be performed for future randomised controlled trials.

## Results

### Recruitment and allocation

**Fig. 2:**
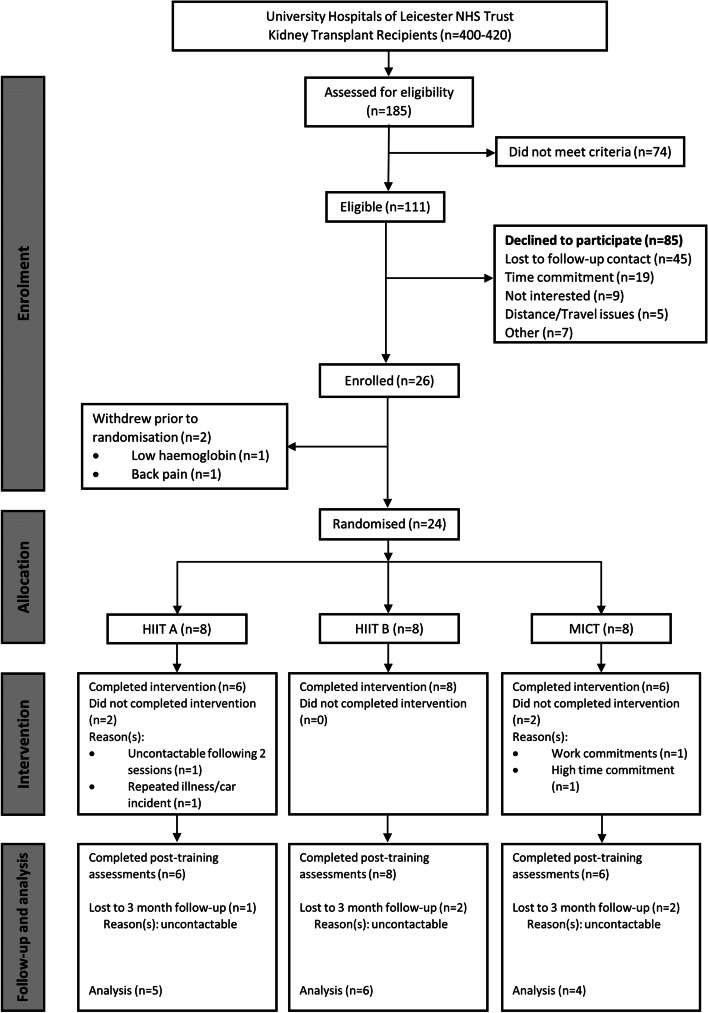
Flow of participants through the trial

Participants were recruited between March 2017 and May 2019. The trial was stopped at the end of the grant funding with the minimum recruitment target being achieved. There are approximately 400–420 KTRs registered at University Hospitals of Leicester NHS Trust. Of the 185 patients who were assessed for eligibility, 111 were eligible, 26 were consented, and 24 were randomised (Fig. 2). The most common reasons for declining to participate (*n* = 85) were uncontactable following 48 h consideration period (*n* = 45), not interested in participating (*n* = 19), and the time commitment was too great (*n* = 9). Eight participants were allocated to each intervention.

### Baseline characteristics

Baseline characteristics are shown in Table 2. Over half of participants (67%) were male, of white ethnicity (75%), and had a mean age of 48 ± 13 years. A lower proportion of males were seen in HIIT A (38%) compared to HIIT B (75%) and MICT (88%). Median transplant vintage (months) was lowest in HIIT A compared to HIITB and MICT (12 ± 20 vs. 44 ± 86 vs. 44 ± 35). Weight and V̇O_2peak_ were highest in MICT (86.5 ± 15.5 kg and 27.5 ± 8.73 mL/kg^-1^/min^-1^, respectively) compared to HIIT A (68.5 ± 15.6 kg and 23.69 ± 6.29 mL/kg^-1^/min^-1^), and HIIT B (84.1 ± 24 kg and 24.65 ± 7.67 mL/kg^-1^/min^-1^).

**Table 2:** Baseline characteristics

Variable	All (*N*=24)	HIIT A (*n*=8)	HIIT B (*n*=8)	MICT (*n*=8)
Age (years)	48 ± 13	41 ± 14	51 ± 11	52 ± 11
Sex (male)	16 (67)	3 (38)	6 (75)	7 (88)
Ethnicity				
White	18 (75)	5 (63)	5 (63)	8 (100)
Indian	3 (13)	2 (25)	1 (13)	0 (0)
White-Black Caribbean	1 (4)	0 (0)	1 (13)	0 (0)
African	1 (4)	0 (0)	1 (13)	0 (0)
Pakistani	1 (4)	1 (13)	0 (0)	0 (0)
Body mass (kg)	79.7 ± 19.7	68.5 ± 15.6	84.1 ± 24	86.5 ± 15.5
BMI	27.2 ± 5.6	25.9 ± 5.4	28.5 ± 7.1	27.1 ± 4.3
V̇O_2 peak_ (L/min)	1.99 ± 0.70	1.56 ± 0.30	2.09 ± 0.86	2.33 ± 0.65
V̇O_2 peak_ (mL/kg^-1^/min^-1^)	25.28 ± 7.48	23.69 ± 6.29	24.65 ± 7.67	27.5 ± 8.73
Systolic blood pressure (mmHg)	133 ± 14	127 ± 11	135 ± 17	137 ± 13
Diastolic blood pressure (mmHg)	85 ± 9	86 ± 6	81 ± 6	88 ± 11
eGFR (mL/min/1.73 m^2^)	58 ± 19	62 ± 18	57 ± 22	55 ± 19
Serum creatinine (mmol/L)	125 ± 49	104 ± 27	133 ± 60	138 ± 53
Kidney transplant vintage (months)^a^	35 ± 52	12 ± 20	44 ± 86	44 ± 35
Medication				
CNI	24 (100)	8 (100)	8 (100)	8 (100)
Steroid	11 (46)	6 (75)	2 (25)	3 (38)
Antihypertensive	22 (92)	7 (88)	7 (88)	8 (100)
Diabetes	5 (21)	1 (13)	3 (38)	1 (13)
Statins	15 (63)	4 (50)	5 (63)	6 (75)

*Abbreviations*: *CNI* calcineurin inhibitor, *eGFR* estimated glomerular filtration rate, *HIIT* high-intensity interval training, *MICT* moderate-intensity continuous training

*Notes*: Unless otherwise indicated, values for categorical variables are expressed as integer (% of *n*); values for continuous variables as mean ± SD. ^a^median (IQR)

### Trial retention and outcome measure completion

Two participants formally withdrew from the trial due to work and time commitments. Two participants were lost to follow-up during the intervention. All 20 participants who completed the intervention attended the post-training assessments. At 3 months post-training, five participants were lost to follow-up (Table 1; 58% trial completion).

For participants who attended assessment sessions the main outcome measure completion was: 98% cardiorespiratory fitness, 95% combined physical function measures, 79% survey packs, and 98% blood sampling.

### Exercise adherence and acceptability

Of the 20 participants who completed the intervention, eight achieved the required exercise intensity in watts (breakdown by group: HIIT A = 0/6 [0%], HIIT B = 3/8 [38%], and MICT = 5/6 [83%]). The mean number of sessions to meet the required intensity was 13 ± 6 sessions. Detailed intensity data is shown in Table 3. Average percentage of HR_peak_ for all three groups during sessions was between 80 and 90%. Fifteen participants met the secondary HR_peak_ criteria for each session (breakdown by group: HIIT A = 4/6 [67%], HIIT B = 5/8 [63%], and MICT = 6/6 [100%]). Participants who completed the intervention attended 92% of sessions (22/24). The mean number of weeks to complete 24 sessions was 10 ± 3. There were 105 cancelled and rescheduled sessions; 68/105 for illness, 33/105 for commitments, and 4/105 for investigator illness. The main illness symptoms reported by participants throughout the intervention were cold/flu related (e.g., sneezing, headache, coughing). Numbers of days during the intervention where cold/flu symptoms were reported were: HIIT A = 14, HIIT B = 7, and MICT = 22. There were no major adverse events reported during the trial. One participant stopped exercising during a session due to pain in the abdominal area which was clinically reviewed and considered unrelated to the exercise. Another participant stopped due to pain caused by a urinary tract infection which was being treated clinically at the time.

**Table 3:** Exercise intensity data

		HIIT A (*n*=6*)*			HIIT B (*n*=8)			MICT (*n*=6)
		High intensity	Active rest	Overall	High intensity	Active rest	Overall	
%W_peak_	(All sessions)	62±7	44±9	54±7	72±10	53±10	64±9	56±4
%W_peak_	(Sessions 1-12)	58±7	44±9	51±8	68±11	51±9	60±10	53±4
%W_peak_	(Sessions 13-24)	66±6	45±9	56±6	77±10	55±10	67±9	60±4
%HR_peak_	(All sessions)	88±5	88±6	88±5	89±10	90±11	90±10	80±8

### Patient feedback

Detailed results of the PSQs can be found in Additional file 2. In summary, participants across all groups were highly satisfied with the exercise programme. There were some concerns across all groups around ability to complete the exercise required and supervision was deemed an important factor for feelings of confidence. Participants across all groups found the exercise enjoyable and beneficial and wanted to carry on exercising after the programme. Key benefits expressed by participants were improved fitness, confidence in ability to exercise, having more energy, and a motivation to continue exercising. Assessments were considered acceptable, with the exception of the survey pack, which some participants felt was too long.

### Physiological measures and survey data

Summary data for physiological measurements are presented in Table 4. Survey data are presented in Additional file 1. Pre-intervention versus post-intervention V̇O_2peak_ (mL/kg^-1^/min^-1^) was 24.28±4.91 versus 27.06±4.82 in HIITA, 24.65±7.67 versus 27.48±8.23 in HIIT B, and 29.33±9.04 versus 33.05±9.90 in MICT. Pre-intervention peak power output (PPO) for HIIT A was 119±22 W for HIIT A, 148±67 W for HIIT B, and 176±34 W for MICT. Post-intervention PPO were 140±22 W,181±80 W, and 206±39 W for HIIT A, HIIT B, and MICT, respectively.

**Table 4:** Physiological measures

Variable	HIIT A	HIIT B	MICT	All
V̇O_2peak_ (L/min)				
Baseline	1.55±0.30	2.09±0.86	2.25±0.62	1.96±0.70
Post-training	1.72±0.29	2.31±0.88	2.53±0.69	2.18±0.74
V̇O_2peak_ (mL/kg^-1^/min^-1^)				
Baseline	24.28±4.91	24.65±7.67	29.33±9.04	25.77±7.25
Post-training	27.06±4.82	27.48±8.23	33.05±9.90	28.81±7.83
Peak power output (W)				
Baseline	119±22	148±67	176±34	148±51
Post-training	140±22	181±80	206±39	176±60
Overall physical activity (ENMO; m*g*)				
Baseline	10.44±2.47	13.40±4.84	10.72±0.75	11.52±3.24
Post-training	11.11±3.79	16.35±6.01	12.28±3.61	13.25±4.85
MVPA (min)				
Baseline	35.84±20.92	59.40±28.95	41.64±6.20	45.63±21.98
Post-training	41.02±18.14	77.83±37.21	43.82±14.36	54.22±29.13
Body mass (kg)				
Baseline	61.64±9.70	77.60±24.60	74.70±7.30	71.51±17.54
Mid-training	61.90±8.55	78.80±25.37	74.98±8.93	72.15±18.07
Post-training	61.62±9.42	78.45±26.20	74.98±8.45	71.91±18.56
3 months post-training	62.16±9.68	79.92±27.23	75.53±9.36	72.83±19.36
BMI (kg/m^2^)				
Baseline	23.58±2.25	25.92±5.97	24.00±2.09	24.63±4.04
Mid-training	23.74±2.07	26.28±6.18	24.03±2.54	24.83±4.22
Post-training	23.64±2.31	26.13±6.53	24.00±2.44	24.73±4.41
3 months post-training	23.88±2.85	26.65±6.93	24.23±2.68	25.08±4.77
Body fat (%)				
Baseline	27.68±8.44	28.08±10.19	20.63±7.10	25.96±8.91
Mid-training	27.08±9.13	28.80±10.83	21.40±8.47	26.25±9.53
Post-training	28.52±9.73	29.68±11.02	20.30±7.30	26.79±9.93
3 months post-training	27.80±10.26	30.02±11.72	20.65±8.22	26.78±10.45
Skeletal muscle mass (kg)				
Baseline	24.62±5.48	31.08±11.40	32.93±2.81	29.42±8.34
Mid-training	25.02±5.34	31.25±11.37	32.68±3.23	29.55±8.24
Post-training	24.42±5.82	30.58±11.33	33.23±2.78	29.23±8.41
3 months post-training	24.70±5.52	31.08±11.53	33.30±2.98	29.55±8.45
Fat free mass (kg)				
Baseline	44.56±9.41	56.02±18.97	59.05±4.12	53.01±14.05
Mid-training	45.14±9.11	56.18±18.90	58.50±4.69	53.12±13.82
Post-training	44.14±10.02	55.25±18.94	59.38±3.92	52.65±14.21
3 months post-training	44.78±9.49	55.73±19.06	59.50±4.37	53.09±14.11
Lean mass (kg)				
Baseline	42.10±8.92	52.72±17.89	55.63±4.00	49.95±13.23
Mid-training	42.66±8.75	52.83±17.83	55.08±4.59	50.04±13.04
Post-training	41.68±9.44	51.82±17.77	55.90±3.80	49.53±13.31
3 months post-training	42.30±9.06	52.43±18.01	56.05±4.22	50.02±13.31
Fat mass (kg)				
Baseline	16.94±5.29	21.58±10.96	15.75±6.48	18.48±8.19
Mid-training	16.70±5.82	22.62±12.17	16.48±7.83	19.01±9.22
Post-training	18.98±9.31	23.30±12.72	15.60±6.85	19.81±10.15
3 months post-training	17.38±7.30	24.18±14.58	16.03±7.81	19.74±10.89
Sit-to-stand 60 (reps)				
Baseline	35±9	37±7	29±9	34±9
Mid-training	40±3	41±11	30±14	38±12
Post-training	45±13	41±11	31±13	40±13
3 months post-training	46±16	43±15	33±11	42±15
Sit-to-stand 5 (sec)				
Baseline	8.59±2.69	7.96±1.27	10.89±3.11	8.95±2.50
Mid-training	7.65±2.01	7.62±2.23	11.04±3.62	8.54±2.86
Post-training	7.25±1.97	6.88±1.70	10.69±4.33	8.02±2.99
3 months post-training	7.04±2.05	6.78±1.97	8.90±2.43	7.43±2.17
Gait Speed (m/s)				
Baseline	1.14±0.10	1.22±0.07	1.12±0.03	1.17±0.08
Mid-training	1.21±0.17	1.29±0.18	1.14±0.06	1.24±0.16
Post-training	1.18±0.16	1.26±0.15	1.17±0.02	1.22±0.14
3 months post-training	1.20±0.13	1.28±0.11	1.18±0.02	1.23±0.11
Calf strength left (kg)				
Baseline	40.84±9.53	78.24±52.93	63.42±31.81	64.65±40.05
Mid-training	40.87±11.36	71.33±53.82	65.43±21.84	62.86±37.56
Post-training	42.87±6.05	75.83±57.38	64.67±19.42	66.78±21.84
3 months post-training	45.21±13.33	78.87±60.01	62.19±3.03	65.14±41.16
Calf strength right (kg)				
Baseline	39.68±11.61	68.93±47.86	55.08±22.13	55.03±22.13
Mid-training	41.36±7.20	74.11±51.99	66.24±11.05	63.76±36.35
Post-training	46.27±4.09	76.65±52.18	64.32±22.99	66.28±37.03
3 months post-training	50.58±11.53	72.91±54.78	67.86±15.61	65.44±37.03
Resting heart rate (beats/min)				
Baseline	77±4	76±4	83±6	78±5
Mid-training	75±8	68±5	74±7	72±7
Post-training	76±8	67±4	78±7	73±7
3 months post-training	73±6	72±5	80±4	74±6
Systolic blood pressure (mmHg)				
Baseline	122±12	131±17	135±12	129±14
Mid-training	123±10	129±22	128±12	127±15
Post-training	122±11	122±13	125±12	123±11
3 months post-training	125±15	131±20	125±7	127±15
Diastolic blood pressure (mmHg)				
Baseline	84±6	79±6	89±10	83±8
Mid-training	80±9	78±4	84±10	80±8
Post-training	80±4	75±6	81±10	78±7
3 months post-training	86±4	80±7	81±11	82±7
Stroke volume (ml/beat)				
Baseline	79.84±7.91	102.41±28.97	102.51±15.96	94.91±22.22
Mid-training	82.81±13.41	118.05±45.48	107.88±9.85	103.59±32.56
Post-training	83.09±13.78	106.18±32.87	106.25±3.93	98.50±23.89
3 months post-training	80.75±14.49	97.94±30.27	98.96±7.49	92.40±21.73
Cardiac output (L/min)				
Baseline	6.15±0.38	7.76±2.29	8.49±1.14	7.41±1.82
Mid-training	6.10±0.60	7.82±2.46	7.86±0.21	7.26±1.73
Post-training	6.22±0.62	7.17±2.47	7.32±0.21	6.89±1.86
3 months post-training	5.80±1.34	7.08±2.47	7.85±0.53	6.86±1.86
Total peripheral resistance (dyn·s·cm^5^)				
Baseline	1263.33±95.53	1113.84±349.26	1018.25±231.25	1138.18±259.98
Mid-training	1296.07±199.15	1096.22±358.49	1024.67±94.46	1143.76±269.16
Post-training	1235.00±129.58	1130.47±529.04	967.25±91.56	1121.79±343.49
3 months post-training	1440.60±324.73	1267.70±470.79	981.17±146.75	1248.92±384.26
Cholesterol (mmol/l)				
Baseline	4.24±0.36	4.11±0.56	4.48±0.97	4.26±0.66
Post-training	4.22±0.91	4.19±0.81	4.12±0.65	4.12±0.65
Total cholesterol/HDL Ratio				
Baseline	3.56±1.07	3.20±1.11	3.33±0.87	3.27±0.95
Post-training	3.10±0.75	3.33±1.14	3.25±0.88	3.22±0.90
Triglyceride (mmol/l)				
Baseline	1.59±0.27	1.03±0.17	2.06±1.49	1.49±0.91
Post-training	1.38±0.43	1.07±0.37	1.71±1.25	1.39±0.76
Creatinine (μmol/L)				
Baseline	107.00±20.25	133.00±60.15	137.17±58.34	126.45±50.10
Post-training	106.00±25.43	132.50±51.88	142.67±64.05	127.60±49.70
Phosphate (mmol/l)				
Baseline	0.87±0.21	1.06±0.25	1.09±0.24	1.01±0.25
Post-training	0.89±0.22	1.09±0.20	1.02±0.25	1.01±0.23
Iron (μmol/L)				
Baseline	17.40±5.94	13.13±4.39	17.67±6.35	15.68±5.62
Post-training	13.00±4.85	13.13±4.36	15.83±3.71	13.95±4.26

*Abbreviations*: *BMI* body mass index, *ENMO* Euclidean norm minus one, *HIIT* high-intensity interval training, *MICT* moderate-intensity continuous training, *MVPA* moderate to vigorous physical activity, *HDL* high density lipoprotein

Data are presented as mean ± SD

### Cardiac MRI (sub-study results)

Seven of the 10 participants invited for a CMR scan completed both pre- and post-intervention scans (5 males; age 47±8 years; eGFR 53±15 mL/min/1.73m^2^; V̇O_2peak_ 26.88±5.67 mL/kg^-1^/min^-1^; PPO 179±45 W). Data are presented in Table 5.

**Table 5:** Cardiac MRI and cardiorespiratory fitness sub-study data

Variable	Baseline (*n*=7†)	Post-training (*n*=7)
*Cardiorespiratory fitness*		
V̇O_2peak_ (L/min)	2.26 ± 0.68	2.50 ± 0.68
V̇O_2peak_ (mL/kg^-1^/min^-1^)	26.88 ± 5.67	29.99 ± 6.99
Peak power output (W)	179 ± 45	207 ± 54
*Cardiac MRI*		
Global native T1 time (ms)	1256.1 ± 53.6	1216.9 ± 31.7
LV mass (g)	134.97 ± 31.85	137.26 ± 25.53
LVEF (%)	63.8 ± 7.9	64.7 ± 7.2
LVM/VEDV (g/ml)	0.85 ± 0.13	0.85 ± 0.12
RVEF (%)	57.5 ± 7.2	55.9 ± 9.9
GLS (%)	-16.2 ± 1.9	-15.9 ± 2.7
GCS (%)	-18.7 ± 1.9	-18.5 ± 3.1
cPEDSR (%^-1^)	0.98 ± 0.18	0.93 ± 0.26
lPEDSR (%^-1^)	0.79 ± 0.21	0.82 ± 0.27

*Abbreviations*: *cPEDSR* circumferential peak early diastolic strain rate, *GCS* global circumferential strain, *GLS* global longitudinal strain, *lPEDSR* longitudinal peak early diastolic strain rate, *LVEF* left ventricular ejection fraction, *LV* left ventricular, *LVEDV* left ventricular end diastolic volume

Data are presented as mean ± SD

## Discussion

To our knowledge, this is the first randomised controlled trial to evaluate the feasibility and acceptability of HIIT in the kidney transplant population. To date, studies in KTRs have focussed on low to moderate-intensity exercise [24, 40]. There were no reported exercise-related adverse events in any group and feedback was generally positive.

Three of our predefined progression criteria were ‘go’ (eligibility, intervention completion in terms of session attendance, and outcome completion). Two criteria, recruitment and trial completion, were acceptable (with modification) at between ‘stop’ and ‘go.’ For patients who were eligible and gave reasons for not wishing to take part, time commitment was the top reason. Future trials may consider reducing the number of supervised days in favour of home-based training or a gradual decline in supervision over time but with consideration to feedback which suggests that supervision was an important contributor to participant confidence. Five participants who completed the intervention did not attend the 3-month follow-up visit which contributed to the lower trial retention figure. This may be mitigated by maintaining contact with participants post-training or providing some continuation of training in a less supervised capacity.

Overall, only 40% of participants achieved the required intensity during the intervention, taking just over half of the intervention duration to achieve this (in HIIT and MICT), suggesting a familiarisation period and longer intervention may be beneficial. More participants in the MICT group achieved their required intensity than in either HIIT group; however, they presented with a higher baseline cardiorespiratory fitness and PPO. Previous studies in heart transplant recipients and people receiving haemodialysis utilising similar HIIT protocols have based their exercise intensity on 85–95% HR_peak_ [28, 41, 42]. Hypertension is thought to affect at least 90% of KTRs [43], with a significant amount requiring anti-hypertensive therapy including beta-blockers which influence heart rate during exercise. Therefore, we applied percentage of peak watts achieved during maximal exercise testing as a primary indicator of intensity to mitigate the potential heart rate heterogeneity. However, had we used % of HR_peak_ as the primary marker of intensity, 75% of the participants would have achieved the required commonly utilised intensity of 85–95% of HR_peak_.

There are several speculative reasons why participants in the HIIT groups were unable to achieve the required intensity (in watts) for the higher bouts; one is that participants did not have sufficient recovery during the lower intensity bouts. Mean heart rates did not drop during the recovery periods. Furthermore, we believe that the use of a continuous ramped protocol for the cardiopulmonary exercise test was perhaps not the most appropriate to dictate the training intensity for this kind of training. While participants were able to achieve these intensities during the test, they were not maintaining them for several minutes repeatedly as in the training protocol.

Calcineurin inhibitors and corticosteroids, which are widely prescribed in KTRs, have been associated with muscle atrophy and decreased muscular oxidative capacity [44]. Peripheral muscle force is reported to be lower in lung transplant recipients compared to those with chronic obstructive pulmonary disease [44], and immunosuppressive medications similar to those taken by KTRs are thought to be a attributing factor [45]. Mathur et al. also postulate an early onset of glycolytic metabolism, potentially explaining the impairment to higher intensity exercise. Low mitochondrial gene expression of PGC-1a, NRF-1, NRF-2, TFam, mfn2, and SOD1/2 has been reported in patients with CKD (non-dialysis) compared to healthy controls [46], which could transpire in KTRs and explain the inability to achieve the intensity required within the intervention timeframe. Participants in HIIT A were far more comfortable with increasing their intensity levels during the final 1-min higher intensity bouts as opposed to the longer 4- and 2-min bouts. Participants were also more likely to want to drop their intensity level during the 4-min bouts, suggesting that shorter more intense intervals may be more acceptable. A large number of sessions were cancelled due to illness; these were mainly cold/flu symptoms which is not surprising due to immunosuppression leaving KTRs susceptible to a broad array of viral pathogens [47]. High-intensity exercise can have an immunosuppressive effect 24 h post exercise [48]; however, reported symptoms were similar between HIIT and MICT. Participants who experienced cold/flu symptoms were reluctant to return to training at the same intensity which could have slowed overall intensity progression.

Limitations include a small sample size due to the trial feasibility nature. We are unable to detect statistical changes due to inadequate powering. However, data can be used to generate sample size calculations for future trials. There is heterogeneity in participant characteristics at baseline with respect to gender, blood pressure, cardiorespiratory fitness, and transplant vintage which may have influenced responses to training. Future trials may consider further stratification or modified inclusion/exclusion criteria. To avoid differences in length of time to complete the intervention, future studies should define a set number of weeks training as opposed to a set number of sessions.

To our knowledge, this is the first study to perform pre- and post-exercise training CMR scans in KTRs. This exploratory study did not raise any safety issues, and there were no signals for adverse effects on traditional measures of cardiovascular structure and function assessed with CMR. Native T1 is a surrogate measure of myocardial fibrosis and inflammation, which are common in patients with CKD [49]. The observed reduction in native T1 times described in this study suggests a possible reduction in levels of myocardial inflammation and fibrosis, but clearly, these results are hypothesis generating and should be further explored in future studies.

Key suggestions for future studies:

- Include a familiarisation/run-in period prior to commencing the training period
- Define a set number of weeks training as opposed to set number or sessions. Twelve weeks could provide a substantial training period and allow for days missed due to illness
- Consider refinement of inclusion criteria to address the impact of factors such as current physical activity levels and kidney transplant vintage. Participants less than one year post transplant may introduce additional considerations that accompany being a new transplant recipient
- Consider further stratification during randomisation to address heterogeneity of sample
- Consider HIIT protocols with shorter intervals as participants may struggle to maintain the required power for 4 min
- Consider less intense recovery periods between high-intensity bouts
- Shorter overall sessions may help address the time commitment barrier to recruitment and barrier to exercise in general
- Consider a stepped protocol for the maximal exercise test which matches the training type rather than a continuous ramp protocol
- Consider a hybrid protocol which incorporates some home-based sessions to help address recruitment

## Conclusions

HIIT and MICT performed on a cycle, with some modification, could be considered safe and feasible in KTRs. Larger scale trials are required to assess the efficacy of HIIT in KTRs and in particular identify the most appropriate intensities, recovery periods, and session duration. Some flexibility in delivery, such as incorporating home-based sessions, may need to be considered to improve recruitment and retention. This study provides evidence that both HIIT and MICT may be useful prescriptions to reduce CVD burden in KTRs.

## Supplementary Information

**Additional file 1.** Survey Pack Data. Results of surveys within the participant survey packs.

**Additional file 2.** Intervention Feedback Data. Data from patient satisfaction questionnaires.

## Data Availability

All data generated or analysed during this study are included in this published article [and its supplementary information files].

## Abbreviations

BMI: Body mass index
CMR: Cardiac magnetic resonance imaging
CVD: Cardiovascular disease
CNI: Calcineurin inhibitor
CKD: Chronic kidney disease
eGFR: Estimated glomerular filtration rate
ENMO: Euclidean norm minus one
HR_peak_: Heart rate peak
HDL: High-density lipoprotein
HIIT: High-intensity interval training
KTR: Kidney transplant recipient
LV: Left ventricular
MICT: Moderate-intensity continuous training
MVPA: Moderate to vigorous physical activity
PSQ: Patient satisfaction questionnaire
PA: Physical activity
PPO: Peak power output
QoL: Quality of life
RPE: Rating of perceived exertion
REC: Research ethics committee
V̇O_2peak_: Oxygen uptake peak
