# Supplementary material for: Feasibility and acceptability of high-intensity interval training and moderate-intensity continuous training in kidney transplant recipients: the PACE-KD study
Source: Pilot Feasibility Stud. 2022 May 21;8:106. doi: 10.1186/s40814-022-01067-3 (PMC9123685; doi:10.1186/s40814-022-01067-3)
Supplement: Supplementary file 1 — Additional file 1. Survey Pack Data. Results of surveys within the participant survey packs. [file 40814_2022_1067_MOESM1_ESM.docx]

**Additional File 1**

**PACE-KD Survey Pack Data**

**Feasibility and acceptability of high intensity interval training and moderate intensity continuous training in kidney transplant recipients: The PACE-KD study**

Roseanne E Billany^1,2^; Alice C Smith^2,3^; Ganisha M Hutchinson^4^; Matthew PM Graham-Brown^1,2^; Daniel GD Nixon^2,3^; Nicolette C Bishop^5^

^1^Department of Cardiovascular Sciences, University of Leicester, Leicester, UK

^2^John Walls Renal Unit, University Hospitals of Leicester NHS Trust, Leicester, UK

^3^Department of Health Sciences, University of Leicester, Leicester, UK

^4^Department of Respiratory Sciences, University of Leicester, Leicester, UK

^5^School of Sport, Exercise and Health Sciences, Loughborough University, Loughborough, UK

Corresponding author: Nicolette C Bishop, Ph.D., School of Sport, Exercise and Health Sciences, Loughborough University, Loughborough, LE11 3TU, United Kingdom; E-mail: [N.C.Bishop@lboro.ac.uk](mailto:N.C.Bishop@lboro.ac.uk).

Table S1. Sleep, fitness, quality of life, and symptoms across groups.

|  |  | HIIT A | HIIT B | MICT | All |
| --- | --- | --- | --- | --- | --- |
| PSQI (0 to 21)^b^ | |  |  |  |  |
|  | Baseline | 5±1 | 5±4 | 5±3 | 5±3 |
|  | Mid-training | 5±1 | 3±1 | 5±2 | 4±2 |
|  | Post-training | 5±2 | 6±1 | 6±3 | 5±2 |
|  | 3 months | 6±2 | 4±3 | 5±2 | 5±2 |
|  |  |  |  |  |  |
| ESS (0 to 24)^b^ | |  |  |  |  |
|  | Baseline | 4±1 | 4±3 | 4±3 | 4±2 |
|  | Mid-training | 3±2 | 4±3 | 3±2 | 3±2 |
|  | Post-training | 2±2 | 4±4 | 4±1 | 4±3 |
|  | 3 months | 3±3 | 6±5 | 3±2 | 4±3 |
|  |  |  |  |  |  |
| DASI (2.74 to 9.89 METs)^a^ | |  |  |  |  |
|  | Baseline | 8.34±1.11 | 9.19±1.03 | 9.28±0.53 | 8.95±0.99 |
|  | Mid-training | 9.33±0.85 | 9.38±0.48 | 9.58±0.53 | 9.40±0.61 |
|  | Post-training | 8.96±0.67 | 9.74±0.41 | 9.20±0.46 | 9.33±0.59 |
|  | 3 months | 8.96±0.78 | 9.66±0.46 | 9.89±0.00 | 9.47±0.65 |
|  |  |  |  |  |  |
| EQ-5D Index (-0.285 to 1)^a^ | |  |  |  |  |
|  | Baseline | 0.94±0.07 | 0.94±0.07 | 0.96±0.07 | 0.94±0.06 |
|  | Mid-training | 0.90±0.10 | 0.95±0.10 | 0.96±0.07 | 0.94±0.09 |
|  | Post-training | 0.90±0.10 | 1.00±0.00 | 0.97±0.06 | 0.95±0.07 |
|  | 3 months | 0.90±0.07 | 1.00±0.00 | 0.95±0.09 | 0.95±0.07 |
|  |  |  |  |  |  |
| EQ-5D Health (0-100 %)^a^ | |  |  |  |  |
|  | Baseline | 80±12 | 73±17 | 78±08 | 76±13 |
|  | Mid-training | 79±13 | 83±10 | 80±08 | 81±10 |
|  | Post-training | 84±09 | 86±07 | 76±15 | 83±13 |
|  | 3 months | 76±15 | 88±06 | 82±03 | 83±11 |
|  |  |  |  |  |  |
| POS-S Symptom Numbers (0-28)^b^ | |  |  |  |  |
|  | Baseline | 6±3 | 7±9 | 7±7 | 7±6 |
|  | Mid-training | 5±4 | 7±7 | 3±2 | 5±5 |
|  | Post-training | 6±4 | 3±4 | 6±3 | 4±4 |
|  | 3 months | 8±2 | 4±6 | 7±5 | 6±5 |

*Abbreviations: DASI, Duke Activity Status Index; ESS, Epworth Sleepiness Scale; EQ-5D, European Quality of Life*

*Group – 5D; MET, metabolic equivalent; POS-S, Palliative Care Outcome Scale; PSQI, Pittsburgh Sleep Quality Index*

*Notes: ^a^Higher Score is better; ^b^Lower score is better*

Table S2. Illness Perception Questionnaire – Revised across groups

|  |  | HIIT A | HIIT B | MICT | All |
| --- | --- | --- | --- | --- | --- |
| IPQ-R Identity (0-14) | |  |  |  |  |
|  | Baseline | 6±3 | 1±1 | 3±4 | 3±3 |
|  | Mid-training | 5±5 | 2±3 | 4±4 | 4±4 |
|  | Post-training | 3±4 | 2±2 | 5±4 | 3±3 |
|  | 3 months | 3±3 | 3±2 | 3±4 | 3±3 |
|  |  |  |  |  |  |
| IPQ-R Timeline (acute/chronic) (0-30) | |  |  |  |  |
|  | Baseline | 25±5 | 24±5 | 29±1 | 26±4 |
|  | Mid-training | 26±5 | 26±2 | 27±4 | 26±3 |
|  | Post-training | 27±3 | 23±7 | 27±3 | 26±5 |
|  | 3 months | 26±3 | 27±4 | 23±1 | 26±3 |
|  |  |  |  |  |  |
| IPQ-R Consequences (0-30) | |  |  |  |  |
|  | Baseline | 20±5 | 17±8 | 19±7 | 18±6 |
|  | Mid-training | 19±6 | 18±8 | 17±7 | 18±6 |
|  | Post-training | 18±6 | 19±4 | 20±6 | 19±5 |
|  | 3 months | 17±6 | 19±6 | 16±6 | 17±5 |
|  |  |  |  |  |  |
| IPQ-R Timeline Cyclical (0-20) | |  |  |  |  |
|  | Baseline | 10±4 | 8±5 | 9±6 | 9±4 |
|  | Mid-training | 9±4 | 8±3 | 10±5 | 9±4 |
|  | Post-training | 10±3 | 10±3 | 11±2 | 10±3 |
|  | 3 months | 10±4 | 8±1 | 9±5 | 9±3 |
|  |  |  |  |  |  |
| IPQ-R Personal Control (0-30) | |  |  |  |  |
|  | Baseline | 22±4 | 21±4 | 23±4 | 23±6 |
|  | Mid-training | 21±2 | 26±3 | 21±7 | 23±4 |
|  | Post-training | 20±2 | 22±5 | 22±4 | 22±4 |
|  | 3 months | 23±2 | 23±4 | 20±7 | 22±4 |
|  |  |  |  |  |  |
| IPQ-R Treatment Control (0-25) | |  |  |  |  |
|  | Baseline | 15±4 | 18±5 | 17±1 | 17±4 |
|  | Mid-training | 14±2 | 13±2 | 13±4 | 14±2 |
|  | Post-training | 17±1 | 17±4 | 18±3 | 17±3 |
|  | 3 months | 18±2 | 16±3 | 16±3 | 16±3 |
|  |  |  |  |  |  |
| IPQ-R Illness Coherence (0-25) | |  |  |  |  |
|  | Baseline | 22±3 | 22±4 | 22±5 | 22±3 |
|  | Mid-training | 19±2 | 20±4 | 20±5 | 20±3 |
|  | Post-training | 20±4 | 21±4 | 20±3 | 21±4 |
|  | 3 months | 20±4 | 22±3 | 21±4 | 21±3 |
|  |  |  |  |  |  |
| IPQ-R Emotional Representations (0-30) | |  |  |  |  |
|  | Baseline | 20±7 | 13±5 | 13±9 | 15±6 |
|  | Mid-training | 18±6 | 14±5 | 15±9 | 16±6 |
|  | Post-training | 18±6 | 17±4 | 16±7 | 17±5 |
|  | 3 months | 19±5 | 15±1 | 15±6 | 16±4 |

*Abbreviations: IPQ-R, illness perception questionnaire revised*

*Notes: High scores on the identity, timeline, consequences, and cyclical dimensions represent strongly held beliefs about the number of symptoms attributed to the illness, the chronicity of the condition, the negative consequences of the illness, and the cyclical nature of the condition*

*High scores on the personal control, treatment control and coherence dimensions, represent positive beliefs about the controllability of the illness and a personal understanding of the condition*

Illness Perception Questionnaire – Revised

Table S3. Baseline top 3 [beliefs of] causes of kidney problem (n=19)

| 1 | 2 | 3 |
| --- | --- | --- |
| genetic defect | bad luck | slow detection when an infant |
| born with the condition | | |
| chronic renal failure | obstructed kidney | |
| blocked ureter | |  |
| hereditary | late diagnosis | smoker |
| reflux | natural degrading | mis-diagnoses |
| hereditary | age | diet |
| hereditary | drugs | not taking medication |
| hereditary | stress | emotional state |
| genetics |  |  |
| hereditary | unlucky | stress |
| hereditary | diabetes | chance |
| hereditary | diet | chance |
| poor medical care | virus/germ | environment |
| uncontrolled blood pressure | diet | health habits |
| over worked | poor medical care in past | environment |
| hypertension | high potassium intake | holding urine for longer durations |
| hereditary | |  |
| stress | mental attitude | family problems |

Palliative Care Outcome Scale Renal (POS-S Renal)

Table S4. Baseline POS-S data


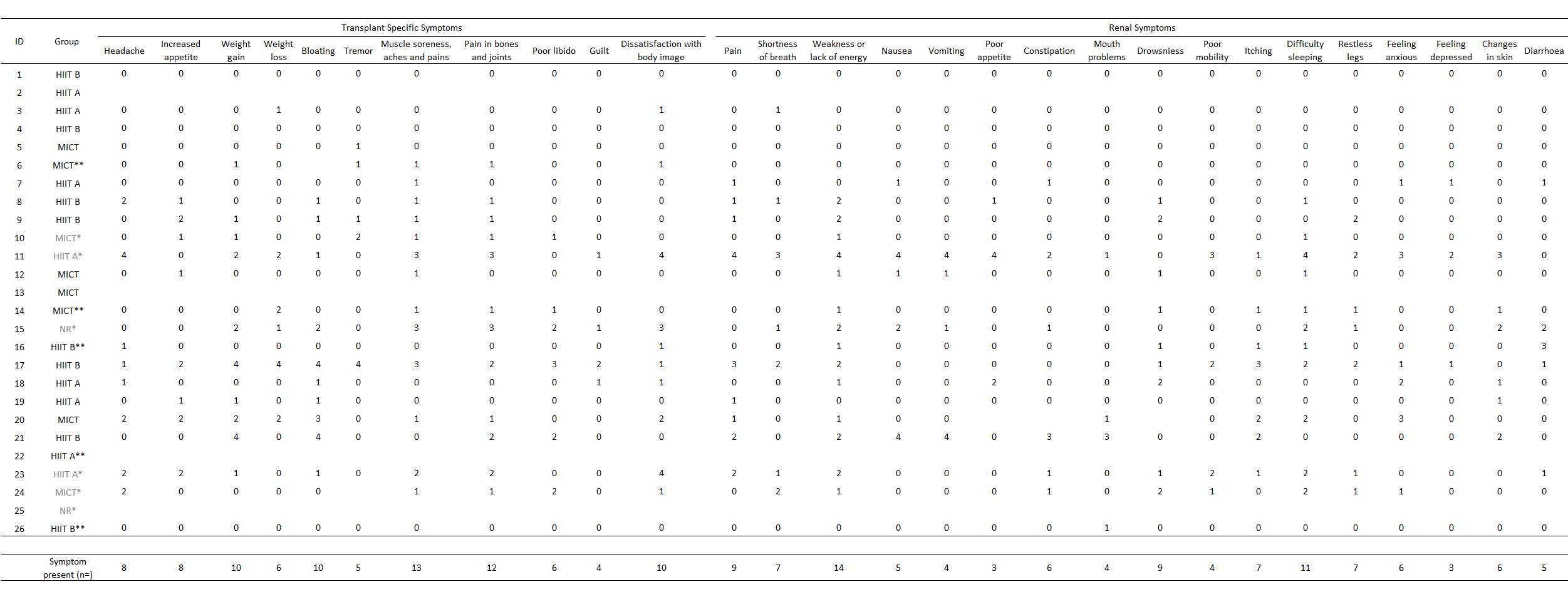


*Abbreviations: HIIT, high intensity interval training; MICT, moderate intensity continuous training; NR, not randomised*

*Notes: *left trial or NR; **did not attend one or more time point; 0-4 relate to symptom affect over the previous week, 0=not at all, 1=slightly, 2=moderately, 3=severely, 4=overwhelmingly*


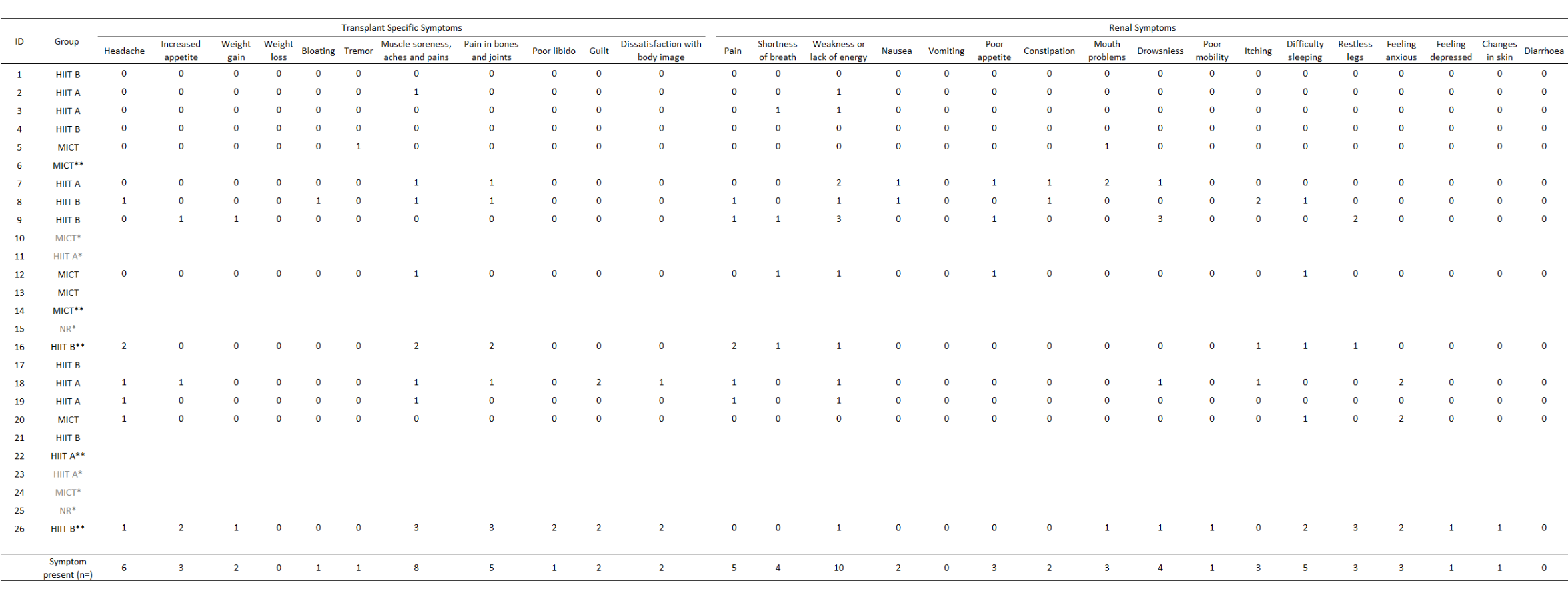
Table S5. Mid-training POS-S data

*Abbreviations: HIIT, high intensity interval training; MICT, moderate intensity continuous training; NR, not randomised*

*Notes: *left trial or NR; **did not attend one or more time point; 0-4 relate to symptom affect over the previous week, 0=not at all, 1=slightly, 2=moderately, 3=severely, 4=overwhelmingly*

Table S6. Post-training POS-S data


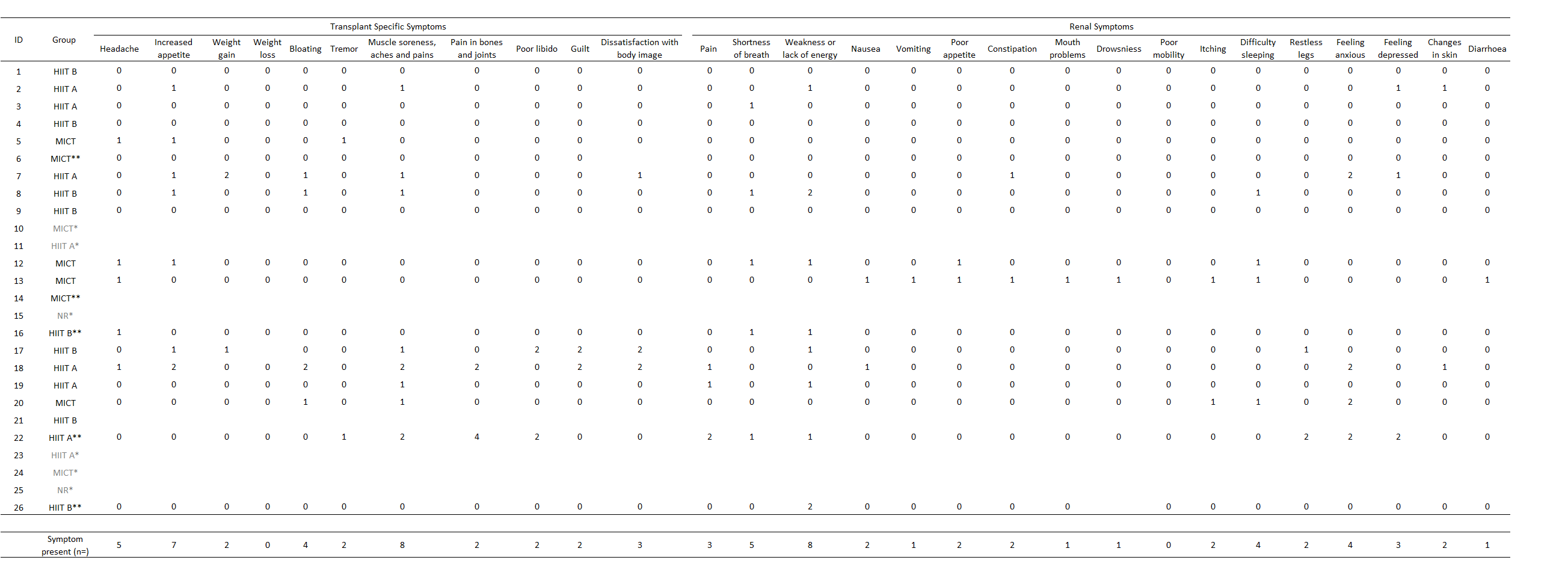


*Abbreviations: HIIT, high intensity interval training; MICT, moderate intensity continuous training; NR, not randomised*

*Notes: *left trial or NR; **did not attend one or more time point; 0-4 relate to symptom affect over the previous week, 0=not at all, 1=slightly, 2=moderately, 3=severely, 4=overwhelmingly*

Table S7. 3 month post-training POS-S data


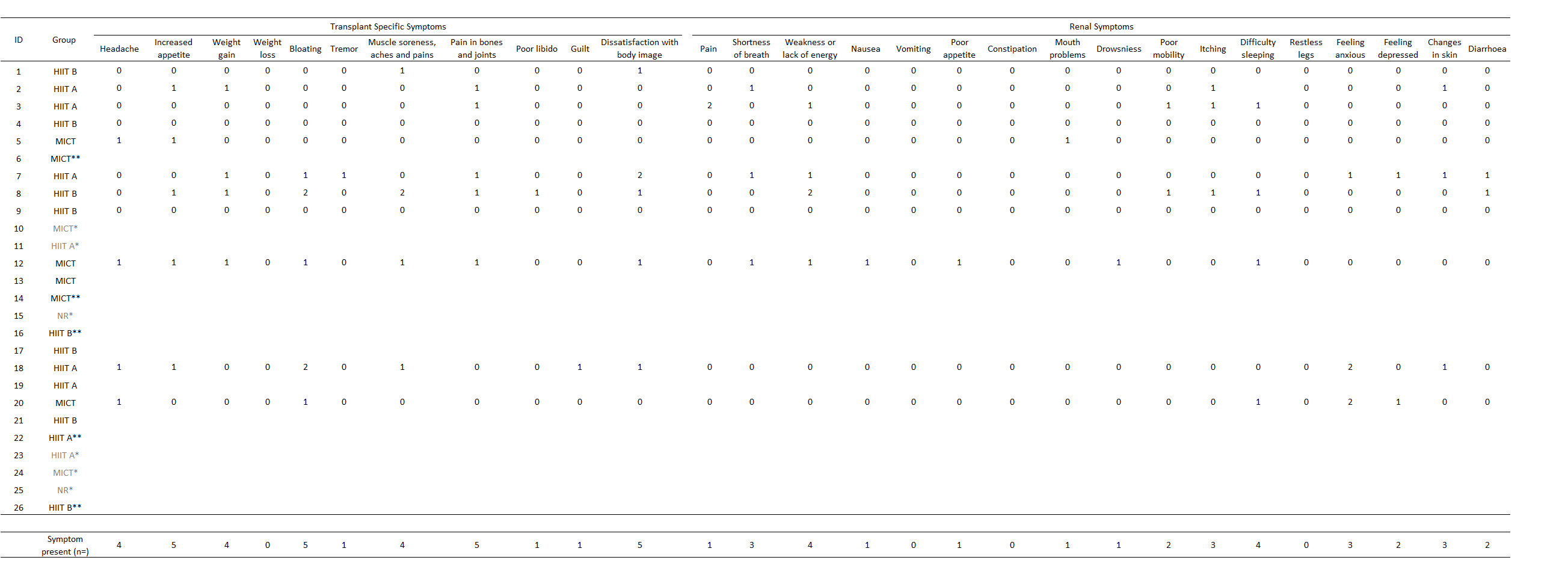


*Abbreviations: HIIT, high intensity interval training; MICT, moderate intensity continuous training; NR, not randomised*

*Notes: *left trial or NR; **did not attend one or more time point; 0-4 relate to symptom affect over the previous week, 0=not at all, 1=slightly, 2=moderately, 3=severely, 4=overwhelmingly*
