# Supplementary material for: Feasibility and acceptability of high-intensity interval training and moderate-intensity continuous training in kidney transplant recipients: the PACE-KD study
Source: Pilot Feasibility Stud. 2022 May 21;8:106. doi: 10.1186/s40814-022-01067-3 (PMC9123685; doi:10.1186/s40814-022-01067-3)
Supplement: Supplementary file 2 — Additional file 2. Intervention Feedback Data. Data from patient satisfaction questionnaires. [file 40814_2022_1067_MOESM2_ESM.docx]

**Additional File 2**

**PACE KD Intervention Feedback Data**

**Feasibility and acceptability of high intensity interval training and moderate intensity continuous training in kidney transplant recipients: The PACE-KD study**

Roseanne E Billany^1,2^; Alice C Smith^2,3^; Ganisha M Hutchinson^4^; Matthew PM Graham-Brown^1,2^; Daniel GD Nixon^2,3^; Nicolette C Bishop^5^

^1^Department of Cardiovascular Sciences, University of Leicester, Leicester, UK

^2^John Walls Renal Unit, University Hospitals of Leicester NHS Trust, Leicester, UK

^3^Department of Health Sciences, University of Leicester, Leicester, UK

^4^Department of Respiratory Sciences, University of Leicester, Leicester, UK

^5^School of Sport, Exercise and Health Sciences, Loughborough University, Loughborough, UK

Corresponding author: Nicolette C Bishop, Ph.D., School of Sport, Exercise and Health Sciences, Loughborough University, Loughborough, LE11 3TU, United Kingdom; E-mail: [N.C.Bishop@lboro.ac.uk](mailto:N.C.Bishop@lboro.ac.uk).

**PACE KD Intervention Feedback (18 responses)**

| **1. Please think about your general involvement in the PACE-KD study.** To what extent do you agree or disagree with the following statements?  (Number of responses reported per option) | Strongly Disagree | Disagree | Neutral | Agree | Strongly Agree |
| --- | --- | --- | --- | --- | --- |
| The purpose of the PACE-KD study was made clear to me |  |  |  | 4 | 14 |
| HIIT A |  |  |  | 2 | 4 |
| HIIT B |  |  |  | 1 | 6 |
| MICT |  |  |  | 1 | 4 |
| I understood what was going to happen at each visit to the hospital for the study |  |  |  | 5 | 13 |
| HIIT A |  |  |  | 2 | 4 |
| HIIT B |  |  |  | 2 | 5 |
| MICT |  |  |  | 1 | 4 |
| There were too many visits to the hospital involved in the study | 4 | 5 | 8 |  |  |
| HIIT A |  | 3 | 3 |  |  |
| HIIT B | 3 |  | 3 |  |  |
| MICT | 1 | 2 | 2 |  |  |
| I had an opportunity to ask questions about the study and my involvement |  |  |  | 5 | 13 |
| HIIT A |  |  |  | 1 | 5 |
| HIIT B |  |  |  | 2 | 5 |
| MICT |  |  |  | 2 | 3 |
| I was concerned at the start about my physical ability to complete the exercises | 1 | 4 | 4 | 9 |  |
| HIIT A |  |  | 1 | 5 |  |
| HIIT B |  | 4 | 1 | 2 |  |
| MICT | 1 |  | 2 | 2 |  |
| I had concerns during the exercise programme about my physical ability to complete the sessions | 1 | 9 | 2 | 5 | 1 |
| HIIT A |  | 5 |  | 1 |  |
| HIIT B |  | 2 | 1 | 3 | 1 |
| MICT | 1 | 2 | 1 | 1 |  |
| I felt motivated during the study to continue exercising |  |  |  | 6 | 12 |
| HIIT A |  |  |  | 2 | 4 |
| HIIT B |  |  |  | 2 | 5 |
| MICT |  |  |  | 2 | 3 |
| I felt adequately supported by the researcher |  |  |  | 4 | 14 |
| HIIT A |  |  |  | 1 | 5 |
| HIIT B |  |  |  | 1 | 6 |
| MICT |  |  |  | 2 | 3 |
| Now I have finished the exercise programme, I want to carry on exercising |  |  |  | 8 | 10 |
| HIIT A |  |  |  | 2 | 4 |
| HIIT B |  |  |  | 3 | 4 |
| MICT |  |  |  | 3 | 2 |

| **2. The PACE-KD Exercise Sessions** | Strongly Disagree | Disagree | Neutral | Agree | Strongly Agree |
| --- | --- | --- | --- | --- | --- |
| I found the exercise sessions enjoyable |  |  | 3 | 6 | 9 |
| HIIT A |  |  |  | 4 | 2 |
| HIIT B |  |  | 1 | 1 | 5 |
| MICT |  |  | 2 | 1 | 2 |
| I had a chance to ask any questions about the exercise |  |  |  | 6 | 12 |
| HIIT A |  |  |  | 2 | 4 |
| HIIT B |  |  |  | 2 | 5 |
| MICT |  |  |  | 2 | 3 |
| The supervision during the exercises was important to me |  | 1 | 2 | 9 | 6 |
| HIIT A |  |  |  | 3 | 3 |
| HIIT B |  | 1 | 1 | 3 | 2 |
| MICT |  |  | 1 | 3 | 1 |
| The supervision made me feel more confident |  |  | 3 | 6 | 9 |
| HIIT A |  |  |  | 2 | 4 |
| HIIT B |  |  | 2 | 1 | 4 |
| MICT |  |  | 1 | 3 | 1 |
| The exercises were varied enough to keep me interested |  | 3 | 8 | 5 | 2 |
| HIIT A |  | 1 | 1 | 3 | 1 |
| HIIT B |  | 2 | 3 | 1 | 1 |
| MICT |  |  | 4 | 1 |  |
| The researchers were motivating throughout the exercise sessions |  | 1 | 1 | 6 | 10 |
| HIIT A |  |  |  | 2 | 4 |
| HIIT B |  | 1 | 1 | 1 | 4 |
| MICT |  |  |  | 3 | 2 |
| The exercise sessions were too long | 4 | 11 | 2 | 1 |  |
| HIIT A | 1 | 3 | 2 |  |  |
| HIIT B | 2 | 5 |  |  |  |
| MICT | 1 | 3 |  | 1 |  |
| The exercise has benefited me |  |  |  | 6 | 12 |
| HIIT A |  |  |  | 1 | 5 |
| HIIT B |  |  |  | 3 | 4 |
| MICT |  |  |  | 2 | 3 |

| ***3. Please think now about, the assessments that you did at the start of the study, in the middle of the study and at the end of the study.***  ***If you have not completed any of the following assessments, please tick ‘not applicable’ on the relevant statements****.* | **Not Applicable** | Strongly Disagree | Disagree | Neutral | Agree | Strongly Agree |
| --- | --- | --- | --- | --- | --- | --- |
| The assessments were clearly explained |  |  |  |  | 9 | 9 |
| HIIT A |  |  |  |  | 4 | 2 |
| HIIT B |  |  |  |  | 3 | 4 |
| MICT |  |  |  |  | 2 | 3 |
| There were too many assessments |  | 5 | 9 | 4 |  |  |
| HIIT A |  | 1 | 4 | 1 |  |  |
| HIIT B |  | 3 | 2 | 2 |  |  |
| MICT |  | 1 | 3 | 1 |  |  |
| The assessments took too long |  | 3 | 9 | 6 |  |  |
| HIIT A |  |  | 3 | 3 |  |  |
| HIIT B |  | 2 | 3 | 2 |  |  |
| MICT |  | 1 | 3 | 1 |  |  |
| The tests helped me to see my progress |  |  | 1 | 3 | 10 | 4 |
| HIIT A |  |  |  | 1 | 5 |  |
| HIIT B |  |  | 1 |  | 3 | 3 |
| MICT |  |  |  | 2 | 2 | 1 |
| I felt I could cope with the VO_2_ Max test |  |  | 2 | 1 | 10 | 5 |
| HIIT A |  |  |  |  | 5 | 1 |
| HIIT B |  |  | 1 | 1 | 2 | 3 |
| MICT |  |  |  | 1 | 3 | 1 |
| I understood why each assessment was important |  |  |  |  | 11 | 7 |
| HIIT A |  |  |  |  | 4 | 2 |
| HIIT B |  |  |  |  | 3 | 4 |
| MICT |  |  |  |  | 4 | 1 |
| I was given the opportunity to split assessments over more than one visit | 1 |  | 2 | 4 | 5 | 6 |
| HIIT A |  |  |  | 2 | 2 | 2 |
| HIIT B | 1 |  | 1 | 1 | 2 | 3 |
| MICT |  |  | 1 | 1 | 1 | 1 |
| The questionnaires I completed with the exercise team were too complicated | 4 | 6 | 6 | 1 | 1 |  |
| HIIT A | 2 | 3 | 1 |  |  |  |
| HIIT B | 2 | 1 | 3 |  | 1 |  |
| MICT |  | 2 | 2 | 1 |  |  |
| The questionnaires I completed with the exercise team took too long | 4 | 4 | 4 | 4 | 2 |  |
| HIIT A | 2 | 2 | 1 |  | 1 |  |
| HIIT B | 2 | 1 | 1 | 2 | 1 |  |
| MICT |  | 1 | 2 | 2 |  |  |

**4a – Are there any other types or additional exercises that you would like to have included in your session workouts?**

**Group A = HIIT A; B = HIIT B; C = MICT**

| ID | Group | Response |
| --- | --- | --- |
| 1 | B | Yes, one more different |
| 2 | A | Rowing machine |
| 3 | C | Running |
| 4 | A | Variation of the exercises |
| 5 | A | Not that I can think of |
| 6 | B |  |
| 7 | B | Running or walking |
| 8 | C |  |
| 9 | C |  |
| 10 | C | I enjoyed the cycling but if other cardio exercises were incorporated in the gym it would have given the program more variety |
| 11 | B |  |
| 12 | B | Treadmill or rowing machine |
| 13 | A |  |
| 14 | C | High intensity training but [I] understood the need for my training |
| 15 | B | No |
| 16 | A | Other equipment |
| 17 | A | I have considered relaxation/stretching/yoga within my exercise training to assist with control of breathing |
| 18 | B | Stretching/ cool down off the bike equipment |

**4b – Do you feel like the study benefited you in any way? If yes, please explain.**

**Group A = HIIT A; B = HIIT B; C = MICT**

| ID | Group | Response |
| --- | --- | --- |
| 1 | B | Yes, feel much better; much lighter and more stamina, healthier. Hoping to continue in daily life. Understood exercise much better |
| 2 | A | Yes, I feel more energetic. Fitter than I started more motivated in physical abilities |
| 3 | C | Yes has improved my fitness. Got me thinking about my general fitness |
| 4 | A | Helped me train muscles, get fitter |
| 5 | A | The study has given me the confidence to partake in exercise and to know that I can safely exercise without any harm to me |
| 6 | B | To push harder when I’m at the gym |
| 7 | B | I wanted to lose weight so this a start |
| 8 | C | Yes it motivated me |
| 9 | C | Yes, motivated me to continue exercise |
| 10 | C | Definitely. Heart rate came down. Felt fitter. Woke up a need to exercise more |
| 11 | B | Yes. Fitness, understanding of my limits and progress made |
| 12 | B | Yes, my cardiovascular fitness improved |
| 13 | A | Yes, love more energy and considering buying treadmill/bike for home |
| 14 | C | Yes. The regular exercise |
| 15 | B | Realistic you can train harder |
| 16 | A | Improved breathing and gave me more confidence to exercise more |
| 17 | A | Yes. Motivation working with researchers kept me going. I am not so good on my own at pushing. I now feel muscles and feel after burn in mind and physically |
| 18 | B | Yes, developing a regular cardiovascular pattern |

**4c – What did you find the hardest thing about the exercise intervention?**

**Group A = HIIT A; B = HIIT B; C = MICT**

| ID | Group | Response |
| --- | --- | --- |
| 1 | B | Commuting a bit, taken more time. Still enjoyed it |
| 2 | A | All forms of exercise are meant to be difficult in order to benefit. I was comfortable |
| 3 | C | Getting into pattern or when get comfortable you go up a level |
| 4 | A | Trying to level up, going to the next difficulty |
| 5 | A | Midway through the exercise study I had a lot of others thing on and I feel that my mental wellbeing affected my participation in the study. After a week on holiday felt more energized and in a better place to participate |
| 6 | B | Committing myself + working around my job |
| 7 | B | Starting exercise again and fitting in all the session |
| 8 | C | Getting to Leicester |
| 9 | C | The first few sessions – getting used to exercising |
| 10 | C | Mentally: from beginning to end. A nagging doubt that I could finish the session |
| 11 | B | Sometimes the exercise was very hard plus blood tests never fun |
| 12 | B | Doing late sessions followed by early sessions |
| 13 | A | My very first session at level 2 |
| 14 | C | The time at constant speed |
| 15 | B | Keeping to the constant speed |
| 16 | A | Maintaining the levels |
| 17 | A | I am not used to being pushed. My motivators have done a great job at this. These sessions have taken the place of my usual exercise and it has been a good change |
| 18 | B | Doing back to back sessions – which was my own choice |

**4d – What did you find the most enjoyable about the exercise intervention?**

**Group A = HIIT A; B = HIIT B; C = MICT**

| ID | Group | Response |
| --- | --- | --- |
| 1 | B | Looking forward to coming and finding the result after exercises. Feeling better |
| 2 | A | The commitment and the workout |
| 3 | C |  |
| 4 | A | Conversations with the researchers |
| 5 | A | I enjoyed having {researcher} there as support during my sessions and the jump from level to level. I enjoyed seeing my progress – shown in the increase of levels |
| 6 | B | The support of the research team |
| 7 | B | I liked the cycling a lot |
| 8 | C | Meeting people who were doing the study work |
| 9 | C | Being able to talk to research – it made time go quicker |
| 10 | C | Seeing and feeling the improvements. Finishing each session |
| 11 | B | Increased fitness and understanding |
| 12 | B | Feeling accomplishment at completing session and team motivation |
| 13 | A | To be honest, I enjoyed all part of the study, feeling like you have become better. |
| 14 | C | The max test |
| 15 | B | At the end of the session having kept to the required speed |
| 16 | A | The support from the research team |
| 17 | A | The feeling at the end of the session to know that I have completed the session and that there was improvement each week |
| 18 | B | Seeing myself improve session by session |

**4e – What could we have done to improve your experience of the exercise program?**

**Group A = HIIT A; B = HIIT B; C = MICT**

| ID | Group | Response |
| --- | --- | --- |
| 1 | B | I have enjoyed thoroughly, because I didn’t have any commitments. Also, lovely staff encouraged me a lot. I have benefited. |
| 2 | A | Variety – cycling, rowing, treadmill |
| 3 | C | Maybe put in another exercise machine. Running so not just on bike all sessions a week |
| 4 | A |  |
| 5 | A | I feel that throughout the study I was always informed and kept updated about things and there was nothing I can think of that would have improved my experience. |
| 6 | B | Longer sessions |
| 7 | B |  |
| 8 | C |  |
| 9 | C |  |
| 10 | C | Nothing really |
| 11 | B | Nothing realistic really. |
| 12 | B | Add music to sessions |
| 13 | A | Wine at end of session |
| 14 | C | nothing |
| 15 | B | nothing |
| 16 | A | Nothing |
| 17 | A | Nothing, it has been great, and I will miss the session. The gym was great, Towel was great, and the heart monitor was comforting |
| 18 | B |  |

**4g – Please provide us with any further comments you may have regarding the assessments or exercise sessions:**

**Group A = HIIT A; B = HIIT B; C = MICT**

| ID | Group | Response |
| --- | --- | --- |
| 1 | B | No comments. I have enjoyed all sessions. Grateful to the staff |
| 2 | A | I am grateful to {researcher} and the team for their support and accommodating me and advice |
| 3 | C |  |
| 4 | A |  |
| 5 | A | I found the cardiac MRI extremely interesting and really enjoyed the information given after this. {doctor} took his time explaining things and was always more willing to answer any questions I had |
| 6 | B |  |
| 7 | B |  |
| 8 | C | I enjoyed it and got into a routine |
| 9 | C |  |
| 10 | C |  |
| 11 | B | Just great really. Top work |
| 12 | B |  |
| 13 | A | I found the team very professional and encouraged me throughout the study |
| 14 | C | Very enjoyable insight into clinical research |
| 15 | B |  |
| 16 | A |  |
| 17 | A | A mix of researcher gave entertainment (good way) as we managed to chat (in some parts) which makes you feel part of the team. |
| 18 | B |  |

**5 – Please rate your overall satisfaction with the exercise programme by circling the appropriate number on the scale below {scale was 1 to 5: results are shown below}**

| **ID** | **1** | **2** | **3** | **4** | **5** | **6** | **7** | **8** | **9** | **10** | **11** | **12** | **13** | **14** | **15** | **16** | **17** | **18** |
| --- | --- | --- | --- | --- | --- | --- | --- | --- | --- | --- | --- | --- | --- | --- | --- | --- | --- | --- |
| **Score** | 5 | 5 | 4 | 4 | 5 | 5 | 4 | 5 | 5 | 5 | 5 | 5 | 5 | 5 | 5 | 5 | 5 | 5 |

**6 – Finally, please use the space below to tell us if:**

Anything about the intervention that has particularly disappointed you.

Anything about the intervention had particularly satisfied you.

Anything else that you think this questionnaire has missed.

**Group A = HIIT A; B = HIIT B; C = MICT**

| ID | Group | Response |
| --- | --- | --- |
| 1 | B | Thankful to the people and staff giving me a chance and advised me to join in. {researcher’s names} been good to me. I had to cancel my sessions couple of times, but staff helped me to finish it alternately. |
| 2 | A | The other questionnaires are long. It would be better to fill in the long questionnaires at the beginning and end with one of the staff. Its better to debate, explain some of the questions. |
| 3 | C | It was hard work, but it gave results. With the team being there, you carried on whereas if on own wouldn’t do it and stop. Managed to get to my goals and will continue to exercise as was something I wanted to do but wasn’t motivated. I would like results as go along but I understand why they don’t as could affect outcome. Overall, I feel fitter in myself and can push further than I thought. |
| 4 | A |  |
| 5 | A | I found the team to be friendly and welcoming and nothing was too much trouble. The team was flexible and accommodating regarding session. The exercise sessions have given me the confidence to carry this on. It’s been 5 years since my transplant, and I have always wanted to partake in exercise but never had the confidence |
| 6 | B | Completing the course, the support from the team in pushing me to hit higher levels as would have stayed at a happy level |
| 7 | B |  |
| 8 | C | It showed I could exercise regular. Also, I need to change my work lifestyle to include regular exercise (This hasn’t happened due to work this summer). Reboot after October and start again for me. Loved being involved with the study |
| 9 | C |  |
| 10 | C | It is a commitment and you do feel you are at the hospital a lot. Especially as a renal patient we get our fair share of hospital appointments. However, I am glad I did it. I felt better by the end and I could see the improvements. I hope to continue a certain level of exercise and I am thankful I finished the program. The team are lovely. |
| 11 | B | Loved it |
| 12 | B |  |
| 13 | A | I enjoyed taking part in this study, team were great, warm and welcoming would highly recommend this study to others. I feel healthier and am going to continue exercising at home. |
| 14 | C | Nothing additional to add |
| 15 | B | Setting own goals and realizing them |
| 16 | A | I am not disappointed with the intervention. The intervention has given me the confidence of my current levels of fitness and to carry on exercising |
| 17 | A | Questionnaire has covered all. It would be nice to have an opportunity for further training sessions as a “maintenance” to my fitness. 3 sessions per week was achievable for an 8-week study. |
| 18 | B | I was very impressed with the teams focus and commitment. They remained friendly, professional and accommodating throughout the 8-week study. |
